# Seeing the primary tumor because of all the trees: Cancer type prediction on low-dimensional data

**DOI:** 10.3389/fmed.2024.1396459

**Published:** 2024-08-27

**Authors:** Julia Gehrmann, Devina Johanna Soenarto, Kevin Hidayat, Maria Beyer, Lars Quakulinski, Samer Alkarkoukly, Scarlett Berressem, Anna Gundert, Michael Butler, Ana Grönke, Simon Lennartz, Thorsten Persigehl, Thomas Zander, Oya Beyan

**Affiliations:** ^1^Institute for Biomedical Informatics, Faculty of Medicine and University Hospital Cologne, University of Cologne, Cologne, Germany; ^2^Medical Data Integration Center (MeDIC), Faculty of Medicine and University Hospital Cologne, University of Cologne, Cologne, Germany; ^3^Department of Internal Medicine, Faculty of Medicine and University Hospital Cologne, University of Cologne, Cologne, Germany; ^4^Center for Integrated Oncology Aachen Bonn Cologne Duesseldorf (CIO ABCD), Aachen, Germany; ^5^Institute for Diagnostic and Interventional Radiology, Faculty of Medicine and University Hospital Cologne, University of Cologne, Cologne, Germany; ^6^Department of Data Science and Artificial Intelligence, Fraunhofer FIT, Sankt Augustin, Germany

**Keywords:** oncology, Cancer of Unknown Primary, prediction, real-world data, classification

## Abstract

The Cancer of Unknown Primary (CUP) syndrome is characterized by identifiable metastases while the primary tumor remains hidden. In recent years, various data-driven approaches have been suggested to predict the location of the primary tumor (LOP) in CUP patients promising improved diagnosis and outcome. These LOP prediction approaches use high-dimensional input data like images or genetic data. However, leveraging such data is challenging, resource-intensive and therefore a potential translational barrier. Instead of using high-dimensional data, we analyzed the LOP prediction performance of low-dimensional data from routine medical care. With our findings, we show that such low-dimensional routine clinical information suffices as input data for tree-based LOP prediction models. The best model reached a mean Accuracy of 94% and a mean Matthews correlation coefficient (MCC) score of 0.92 in 10-fold nested cross-validation (NCV) when distinguishing four types of cancer. When considering eight types of cancer, this model achieved a mean Accuracy of 85% and a mean MCC score of 0.81. This is comparable to the performance achieved by approaches using high-dimensional input data. Additionally, the distribution pattern of metastases appears to be important information in predicting the LOP.

## 1 Introduction

The “Cancer of Unknown Primary” syndrome (CUP) is diagnosed if only metastases but no primary tumor can be found (1). Extensive examination and molecular analyzes without the support of AI currently enable predicting the location of the primary tumor (LOP) for 10–20% of CUP patients with an accuracy of 85–90% (2, 3). For these patients, an LOP-specific treatment can be chosen which significantly improves their prognosis.

Historically, about 3–5% of all cancer cases were diagnosed as CUP (4). Due to advances in diagnostics, this rate could be reduced to 1–2% in general, but it is still higher for patients living in areas with rudimentary clinical care (1, 5, 6). Additionally, the CUP syndrome is still among the 10 most common reasons for cancer-related deaths globally (1). Thus, further advances in LOP prediction are needed to improve the prognosis for CUP patients.

AI-driven data analysis can be a key component in achieving this and some promising approaches have already been developed (1, 7–14). They are described in Supplementary material. A major drawback of these related approaches is their dependency on high-dimensional input data measuring the transcriptome, the mutation pattern, or epigenetic features of the metastases. This data is not generated for cancer patients by default. Hence, the approaches introduce additional costs representing a potential translational barrier for clinical practice. In 2021, Lu et al. (15) have shown that the additional costs to generate transcriptomic, genetic, or epigenetic data might not be needed for most CUP cases. Although only using the sex of the patient and whole slide images (WSI) from pathological examinations as input data, they achieve comparably high classification performance in LOP prediction with a convolutional neural network (CNN) approach (15).

Motivated by the success of Lu et al. (15), we examined whether LOP prediction also works for even lower-dimensional data, i.e., to dispense with image files and instead only use a small number of structured clinical features as input data. Since such data is far less dimensional than genome data or images, the complexity of the task is reduced and the decision-making process becomes more comprehensible.

In 10-fold nested cross-validation (NCV) we examined the LOP prediction performance of a random forest (RF) classifier and a gradient boosted trees (GBT) classifier on three different input feature sets compiled from oncological real-world data (RWD) of non-CUP patients at University Hospital Cologne (UHC). An extensive extract transform load (ETL) process accompanied by interdisciplinary decisions ensured highest possible data quality. Comparing our results to the LOP prediction performance achieved by high-dimensional approaches, shows that our tree-based approach on input features such as the age, sex, histological specifications, lab results, and the distribution pattern of metastases can achieve classification performances as high as the complex approaches while being more transparent, accessible, affordable, and explainable. Especially, the distribution pattern of metastases proved to be a valuable source of information for well-performing classification.

## 2 Materials and methods

### 2.1 Data curation

**Figure 1:**
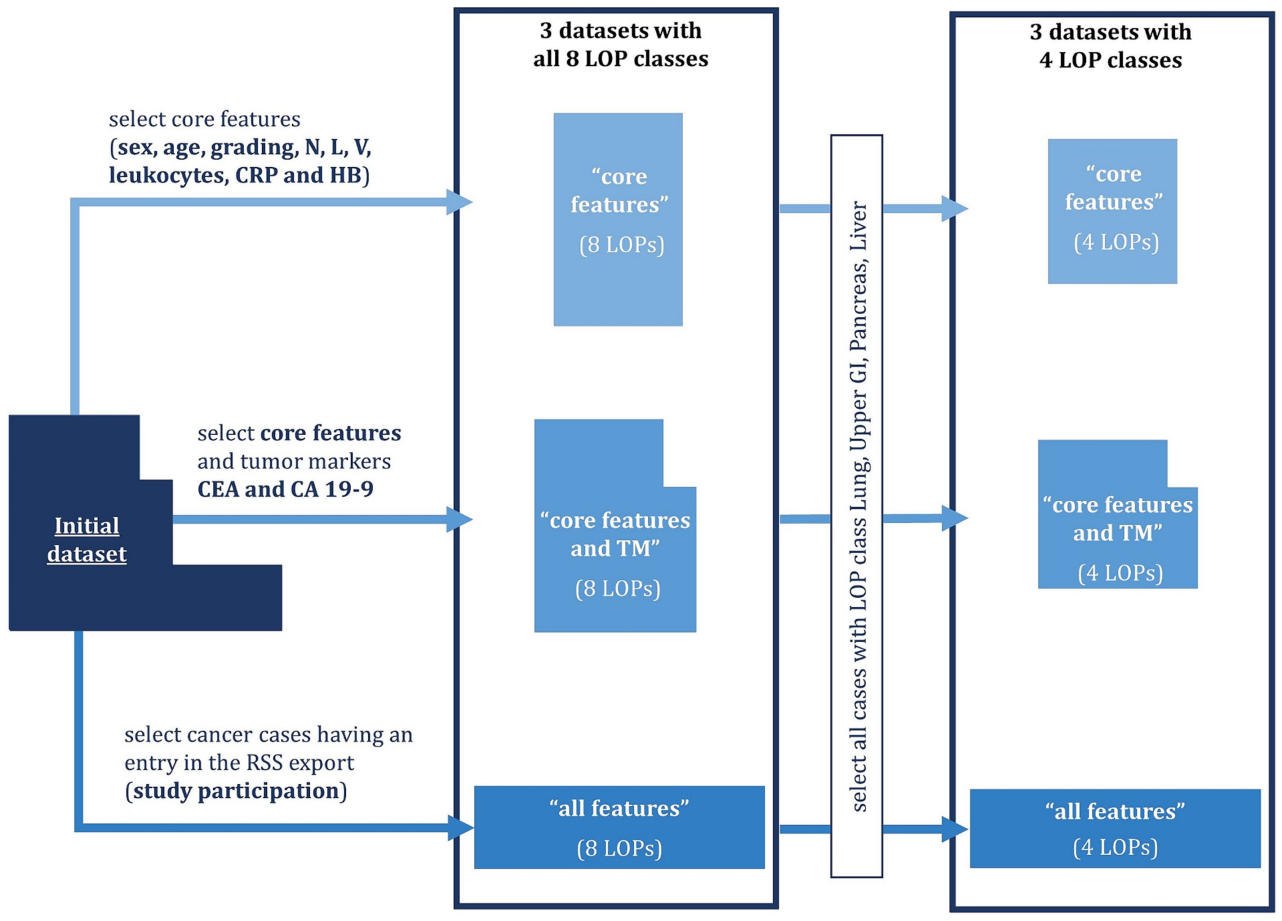
Dataset compilation process. Six datasets (nested boxes) were created which differ in the number of features (width of the boxes) and the number of cases (height of the boxes). The features always contain the core features listed above and optionally tumor markers (TM) as well as organ-specific Tumor Burden Scores (TBS). The number of cases is partly restricted by requiring the case to have an entry in the radiological study system (RSS) or the location of the primary tumor (LOP) to either be Lung, Upper GI, Pancreas, or Liver.

In total, we compiled six datasets from clinical systems of UHC as shown in Figure 1. We included cancer cases of adult patients diagnosed with Lung, Pancreas, Kidney, Liver, Breast, Colorectal, Ears-Nose-Throat, or Upper GI cancer between 01.01.2000 and 30.06.2021. Patients having several cancer diagnoses within 5 years were excluded from the dataset. For each included cancer case, we compiled the age at diagnosis, the sex, histological specification, lab results, and the metastatic burden according to RECIST v1.1 (16). The histological specifications comprised the tumor grading as well as indicators for infestation of lymph nodes (N-value), lymph vessels (L-value) and veins (V-value). The lab results comprised the amount of leukocytes, C-reactive protein (CRP), Hemoglobin (HB), Carbohydrate Antigen 19–9 (CA 19–9), and Carcinoembryonic Antigen (CEA) in the blood. CA 19–9 and CEA are tumor markers (TM), i.e., proteins whose abundance can indicate certain types of tumors. The RECIST evaluations were translated to organ-specific Tumor Burden Scores (TBS) spanning from 0 (no infestation) to 4 (significant infestation). All TBS taken together represent the metastatic distribution pattern by indicating the tumor burden in individual organs. Based on the frequency of missing values for the individual features we created three feature sets:

1. “Core features” containing the age, the sex, histological specifications, leukocytes, CRP, and HB (frequency of missing values below 35%)
2. “Core features and TM” containing the core features and the TM CA 19–9 and CEA (frequency of missing values 77 and 69%, respectively).
3. “All features” containing the core features, the TM, and the organ-specific TBS, which indicate the distribution pattern of metastases (frequency of missing values 98%).

Due to the low availability of the TBS, we only included those cases in the “all features” dataset for which the TBS were available. As a result, four of the eight LOP classes were underrepresented, so we decided to create a four-class version of each dataset only containing the classes that were still well represented: Lung, Upper GI, Pancreas, and Liver. This resulted in a total of six datasets. Missing values were imputed in all six datasets using the R package “mice” in version 3.15.0 for Multiple Imputation by Chained Equations (MICE) (17–20). Eventually, the datasets were anonymized using the software tool ARX, which can anonymize structured data according to a variety of data privacy models (21). In particular, we deleted identifying features and established 5-anonymity with respect to the quasi-identifying features age and sex. This means that we generalized the age to age groups such that at least five patients share the same combination of age and sex. Additionally, ARX suppressed too specific cancer cases that would require huge age groups to achieve 5-anonymity. The sizes of the resulting datasets are depicted in Table 1. More details on the data curation process can be found in Supplementary material.

**Table 1:** Number of cancer cases in the three datasets “core features,” “core features and TM,” and “all features” before (blue) and after (green) anonymization when including all eight classes vs. only including four classes Lung, Upper GI, Pancreas, and Liver.

Number of classes	Cases in “core features” dataset (nine features)		Cases in “core features and TM” dataset (11 features)		Cases in “all features” dataset (30 features)	
	Before anonymization	After anonymization	Before anonymization	After anonymization	Before anonymization	After anonymization
8	13,861	13,764	13,861	13,712	336	328
4	4,295	4,271	4,295	4,271	299	297

### 2.2 LOP prediction

We implemented LOP prediction by classifying the patients according to their type of cancer using a supervised ML approach. In particular, we applied a RF classifier and a GBT classifier on each of the six compiled datasets resulting in 12 classification runs in total. RF and GBT are tree-based ML methods, which have shown good performance in LOP prediction in related work (7–12). An additional advantage of these methods is their inherent explainability, which is a key requirement for AI-based decision support in medical contexts (22, 23). As supervised ML methods, both RF and GBT need class labels throughout model training. In our case, these class labels is the LOP. Therefore, we trained and evaluated the models on medical RWD of cancer patients with known cancer types, i.e., on data of non-CUP patients.

We comprehensively evaluated the performance of the classifiers considering several performance metrics: accuracy, Precision, Recall, F1-score, and MCC score. This performance estimation was combined with 10-fold NCV to decrease the influence of randomness and to determine optimal hyperparameter values for the classifiers from a pre-defined parameter grid. For the RF, the parameter grid contained the values 5, 10, 20, 35, and 50 for the number of decision trees (DTs), the values 3, 5, 7, and 10 for the maximal depth of the DTs, the two entropy measures Gini-Index and Cross-Entropy, as well as training with and without bootstrapping. The parameter grid of the GBT contained the values 0.1, 0.2, and 0.5 for the learning rates and the values 3, 5, 7, and 10 for the maximal depth of the DTs in the GBT sequence. The optimal set of hyperparameter values was chosen by a grid search approach maximizing the MCC score of the classification. We have opted for an optimization according to MCC score due to the high class-imbalance in our datasets and the low sensitivity of the MCC score for such class-imbalances (24). The 10-fold NCV was stratified in order to maintain the class distribution in the test and training dataset. Eventually, we determined the importance of each input feature for LOP prediction based on the average decrease in class entropy over all splits in which the respective feature was the separating feature (25, 26). To enable a systematic comparison of individual features, we determined four groups of features according to their feature importance (FI) for each classification setting, individually: low, medium low, medium high and high FI. The groups were defined based on the quartiles of the FI. More details on the methods and their implementation can be found in Supplementary material.

## 3 Results

### 3.1 LOP prediction performance

**Figure 2:**
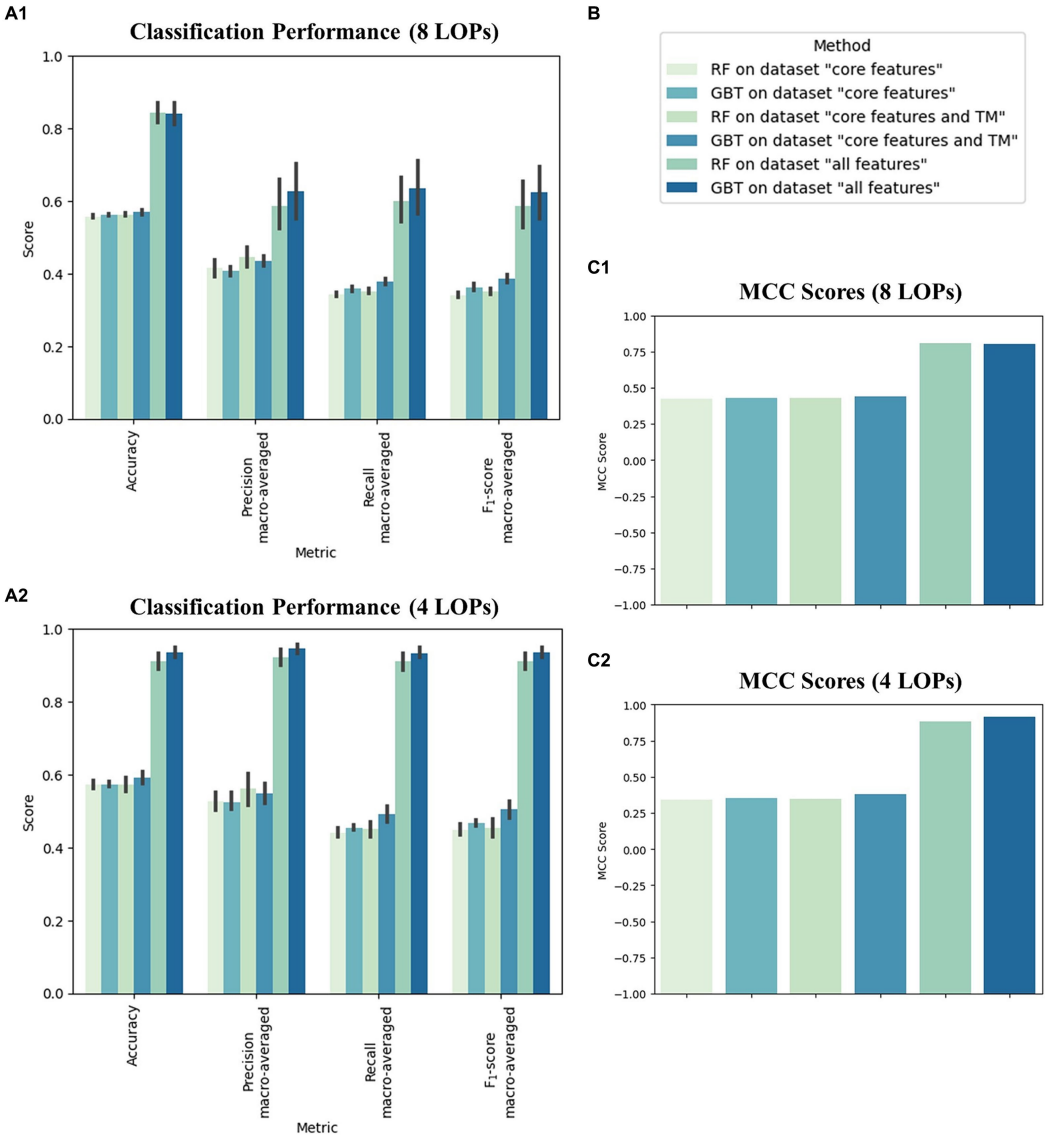
Performance of the two applied machine learning methods random forest (RF) and gradient boosted trees (GBT) on the three feature sets “core features,” “core features and TM” and “all features” in predicting the location of the primary tumor (LOP). **(A)** Average classification performance of the six classifiers across the 10 iterations of the nested cross validation (NCV), measured by Accuracy, macro-averaged Precision, macro-averaged Recall, and macro-averaged F1-score all spanning from 0 to 1 **(B)** Legend displaying assignment of colors to classification settings. **(C)** Average classification performance across the 10 iterations of the NCV measured in terms of the Matthews correlation coefficient (MCC) spanning from −1 to 1. Sections **(A1)** and **(C1)** depict the performance in the eight-class classification task (Breast, Colorectal, ENT, Kidney, Liver, Lung, Pancreas, Upper GI). Sections **(A2)** and **(C2)** depict the performance in the four-class classification task (Lung, Upper GI, Pancreas, Liver).

We applied 10-fold NCV to evaluate the classification performance of the tree-based ML algorithms on the six datasets. Figure 2 shows the mean performance values across the 10 NCV iterations for all examined classification settings, i.e., combinations of algorithm and dataset. In terms of average Accuracy, the performance spanned from 55.8 to 84.5% in the eight-class classification task and from 57.2 to 93.6% in the four-class classification task. The average MCC scores ranged from 0.42 to 0.81 when distinguishing eight LOP classes and from 0.34 to 0.92 when assigning the cancer cases to one of four LOP classes. The achieved performance values were stable across the 10 NCV iterations, which can be seen from the small standard deviations.

For both classification tasks (four and eight LOP classes), we observed that the values of all performance metrics increased with increasing numbers of features. The provision of the TBS (“all features”) led to a particular increase in performance for both ML methods. Moreover, the GBT algorithm exhibited slightly higher performance scores than the RF in almost all combinations of metric and dataset. The only exceptions were the MCC score of the RF on the eight-class “all features” dataset and the Precision of the RF on the “core features” and “core features and TM” datasets. In these settings, the scores were slightly higher for the RF than for the GBT. Another striking observation was that Precision is usually higher than Recall in all classification runs. The only exceptions were the two classifiers trained to discriminate eight LOP classes based on “all features.” These classifiers exhibited a slightly higher Recall than Precision. In general, including the TBS in the input dataset increased both Precision and Recall while decreasing their difference. Thus, including the TBS resulted in a more balanced decision making.

Considering individual combinations of datasets and ML algorithms, we observed that the Accuracy, Precision, Recall, and F1-score are higher in four-class classification than in the eight-class setting. The difference is particularly high on “all features,” i.e., when the TBS are provided. In contrast to the simpler metrics, the MCC score is usually higher in the eight-class classification setting. Only the classification runs on “all features” achieve a higher MCC score when distinguishing between four instead of eight classes.

### 3.2 Feature importance

**Figure 3:**
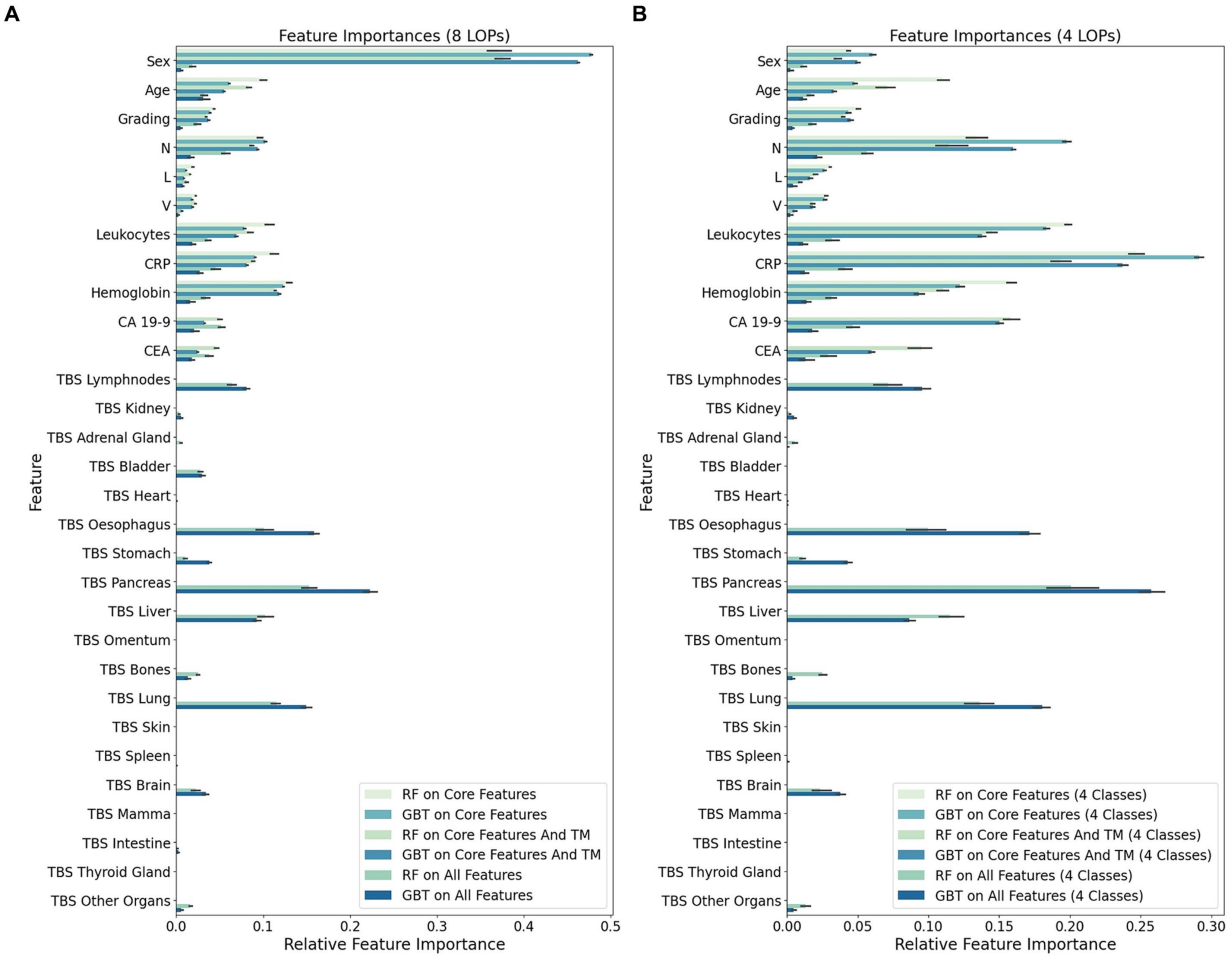
Mean feature importance (FI) for individual input features in 10-fold nested Cross-Validation (NCV). The FI values were determined in every NCV iteration for each combination of machine learning method [random forest (RF) or gradient boosted trees (GBT)] and feature set [“core features” (only first 9 features), “core features and TM” (only first 11 features), or “all features”]. This barplot visualizes the mean FI value of the individual features across 10 NCV iterations. **(A)** Importance of individual features in the eight-class classification task of assigning cancer cases to one of eight LOP classes (Breast, Colorectal, ENT, Kidney, Liver, Lung, Pancreas, Upper GI). **(B)** Importance of individual features in the four-class classification task of assigning cancer cases to one of four LOP classes (Lung, Upper GI, Pancreas, Liver).

For each classification run, i.e., combination of feature set and ML algorithm, we determined the FI of individual features in every NCV iteration. The means of the FI values across NCV iterations are visualized in Figure 3 per feature and classification run.

Particularly striking is the overall decreased importance of the feature sex when not considering the LOP classes Breast, Colorectal, ENT, and Kidney. In this four-class setting, the FI is transferred from sex to all other features having a decent to high importance in the eight-class setting. The gain in FI is particularly high for the features CRP, leukocytes and the N-value. A medium gain can be observed for the other features contained in the feature set “core and TM.” The highest increase in FI among the TBS, which indicate the distribution pattern of metastases, can be seen for the TBS of Pancreas, Lung, Esophagus, and Liver. These TBS features already had a rather high FI in the eight-class setting. The TBS for Brain, Stomach, Bones, and the group of Other Organs were subject to a medium increase in FI.

To enable a more systematic comparison of the FI in the different classification runs, we assigned the features to one of four groups: low, medium low, medium high and high FI. This grouping is based on the first, second, and third quartile of the mean FI value for each classification run and depicted in Table 2.

**Table 2:** Features grouped by their importance for the LOP prediction.

Classification run	Low importance	1st quartile of FI	Medium low importance	2nd quartile of FI	Medium high importance	3rd quartile of FI	High importance
RF on core features (eight LOPs)	Grading (0.043), V (0.022), L (0.019)	0.043	N (0.096)	0.098	Leukocytes (0.107), Age (0.100)	0.110	Sex (0.371), HB (0.130), CRP (0.112)
GBT on core features (eight LOPs)	Grading (0.039), V (0.018), L (0.011)	0.039	Age (0.061)	0.070	CRP (0.091), Leukocytes (0.078)	0.096	Sex (0.477), HB (0.123), N (0.102)
RF on core features and TM (eight LOPs)	Grading (0.034), V (0.022), L (0.016)	0.040	CA 19–9 (0.049), CEA (0.046)	0.066	N (0.087), Leukocytes (0.085), Age (0.083)	0.087	Sex (0.377), HB (0.113), CRP (0.088)
GBT on core features and TM (eight LOPs)	CEA (0.024), V (0.019), L (0.009)	0.028	Grading (0.037), CA 19–9 (0.032)	0.046	CRP (0.081), Leukocytes (0.069), Age (0.055)	0.081	Sex (0.462), HB (0.119), N (0.094)
RF on all features (eight LOPs)	TBS Kidney (0.003), TBS Intestine (0.001), TBS Thyroid Gland (0.000), TBS Mamma (0.000), TBS Spleen (0.000), TBS Skin (0.000), TBS Omentum (0.000), TBS Heart (0.000)	0.004	TBS Brain (0.022), Sex (0.018), TBS Other Organs (0.016), L (0.012), TBS Stomach (0.010), V (0.006), TBS Adrenal Gland (0.005)	0.022	CEA (0.038), Leukocytes (0.036), HB (0.034), Age (0.032), TBS Bladder (0.028), TBS Bones (0.025), Grading (0.024)	0.040	TBS Pancreas (0.153), TBS Lung (0.115), TBS Liver (0.102), TBS Esophagus (0.101), TBS Lymphnodes (0.064), N (0.057), CA 19–9 (0.052), CRP (0.045)
GBT on all features (eight LOPs)	V (0.002), TBS Thyroid Gland (0.000), TBS Mamma (0.000), TBS Spleen (0.000), TBS Skin (0.000), TBS Omentum (0.000), TBS Heart (0.000), TBS Adrenal Gland (0.000)	0.002	TBS Bones (0.013), L (0.007), TBS Other Organs (0.006), Sex (0.006), TBS Kidney (0.005), Grading (0.005), TBS Intestine (0.003)	0.013	TBS Bladder (0.030), CRP (0.027), CA 19–9 (0.021), Leukocytes (0.019), CEA (0.018), HB (0.016), N (0.016)	0.030	TBS Pancreas (0.223), TBS Esophagus (0.158), TBS Lung (0.149), TBS Liver (0.092), TBS Lymphnodes (0.081), TBS Stomach (0.038), TBS Brain (0.034), Age (0.031)
RF on core features (four LOPs)	Sex (0.043), L (0.030), V (0.028)	0.043	Grading (0.050)	0.080	N (0.134), Age (0.110)	0.146	CRP (0.247), Leukocyte (0.199), HB (0.158)
GBT on core features (four LOPs)	Grading (0.043), V (0.027), L (0.027)	0.043	Age (0.048)	0.054	HB (0.122), Sex (0.060)	0.152	CRP (0.291), N (0.198), Leukocyte (0.183)
RF on core features and TM (four LOPs)	Sex (0.035), L (0.02), V (0.018)	0.038	Age (0.071), Grading (0.04)	0.083	N (0.114), HB (0.110), CEA (0.095)	0.114	CRP (0.193), CA 19–9 (0.158), Leukocytes (0.145)
GBT on core features and TM (four LOPs)	Age (0.033), V (0.018), L (0.016)	0.039	Sex (0.05), Grading (0.045)	0.055	Leukocytes (0.138), HB (0.093), CEA (0.06)	0.138	CRP (0.237), N (0.16), CA 19–9 (0.15)
RF on all features (four LOPs)	TBS Spleen (0.001), TBS Thyroid Gland (0.0), TBS Intestine (0.0), TBS Mamma (0.0), TBS Skin (0.0), TBS Omentum (0.0), TBS Heart (0.0), TBS Bladder (0.0)	0.001	TBS Other Organs (0.013), TBS Stomach (0.011), Sex (0.011), L (0.009), TBS Adrenal Gland (0.005), V (0.005), TBS Kidney (0.002)	0.013	Leukocytes (0.032), HB (0.031), CEA (0.029), TBS Bones (0.025), TBS Brain (0.023), Grading (0.018), Age (0.016)	0.034	TBS Pancreas (0.201), TBS Lung (0.136), TBS Liver (0.115), TBS Esophagus (0.1), TBS Lymphnodes (0.072), N (0.057), CA 19–9 (0.047), CRP (0.041)
GBT on all features (four LOPs)	TBS Thyroid Gland (0.0), TBS Intestine (0.0), TBS Mamma (0.0), TBS Spleen (0.0), TBS Skin (0.0), TBS Omentum (0.0), TBS Heart (0.0), TBS Bladder (0.0), TBS Adrenal Gland (0.0)	0.000	TBS Other Organs (0.005), TBS Kidney (0.005), TBS Bones (0.004), L (0.004), Grading (0.004), V (0.002), Sex (0.002)	0.005	CA 19–9 (0.018), HB (0.014), CEA (0.013), CRP (0.013), Leukocytes (0.011), Age (0.011)	0.019	TBS Pancreas (0.257), TBS Lung (0.18), TBS Esophagus (0.171), TBS Lymphnodes (0.096), TBS Liver (0.086), TBS Stomach (0.043), TBS Brain (0.038), N (0.022)

For each classification run, i.e., combination of dataset and ML algorithm, we created four groups of features according to their individual mean feature importance (FI) for the respective classification in 10-fold NCV. The groups were determined based on the first, second and third quartile of the FI among all features in the respective dataset for the respective classification setting: features with a FI at most 1st quartile (low importance), features with a FI above 1st quartile and at most 2nd quartile (medium low importance), features with a FI above 2nd quartile and at most 3rd quartile (medium high importance) and features with a FI above 3rd quartile (high importance). All values shown in this table are rounded to three decimal places.

CRP, leukocytes, HB, the N-value and the age exhibited a high or medium high importance in the majority of classification runs. The feature sex was categorized diversely. When the TBS were not provided, the eight-class classification runs assigned a high importance to the sex while it was of low or medium low importance for almost all four-class classification runs. All approaches on the “all features” dataset categorized the sex to have a medium low importance. The grading had a medium high FI in the RF-based classification runs on the “all features” datasets. All other classification runs assigned a lower importance to it (medium low or low). The L- and the V-value both are categorized to have rather low FI. The TM CA 19–9 and CEA were assigned a rather high importance. Out of eight classification runs using the TM as input features, six categorized CEA to have a medium high and CA 19–9 to have high or medium high FI. The two eight-class runs on the “core and TM” dataset considered CA 19–9 to have a medium low importance and CEA to have a medium low or low importance. In general, CA 19–9 received higher FI scores than CEA.

The TBS were only provided as input features in four out of 10 classifications. In these four classifications, the group of highly important features mainly consists of TBS features. In particular, the TBS for Lymphnodes, Esophagus, Pancreas, Lung and Liver were assigned a high importance for LOP prediction. Only four non-TBS features were categorized as high importance features in a classification run on “all features”: the N-value, CA 19–9, CRP, and the age.

A rather high importance was assigned to the TBS for Brain and Stomach while the TBS for Bladder received diverse categorizations. In the four-class classification runs, the TBS Bladder exhibited low importance for the LOP prediction while it had a medium high FI in the eight-class setting. The TBS for Kidney, Adrenal Gland, and Other Organs were assigned low or medium low FI in all four runs on “all features.” The TBS for Intestine exhibited a medium low importance once. In all other classification runs, it had low importance. In two classifications it even achieved a mean FI of not more than 0. The TBS for Heart, Omentum, Skin, Spleen, Mamma, and Thyroid Gland belong to the features with low importance in all classification runs on “all features.” It is noticeable that, with the exception of TBS Spleen, all these TBS have an average FI value of 0 in all four classifications. This means that the values of these TBS were not considered in any classification.

## 4 Discussion

### 4.1 LOP prediction performance on low-dimensional data

We observed a generally higher LOP prediction performance when considering four instead of eight LOP classes. This was particularly true for the rather simple performance metrics Accuracy, Precision, Recall, and F1-Score. For these metrics, the baseline performance value of a predictor assigning classes randomly is higher with fewer classes. So, we explain the lower values of these metrics in the eight-class setting by the larger number of classes. The MCC score of the LOP prediction is slightly higher in the eight-class setting if the prediction model is provided with the “core features” or the “core features and TM.” Since the difference in MCC scores in the two settings is very small, we consider this to be a random phenomenon. It is made possible by the restricted information content of the “core features” and the “core features and TM” feature sets. On the feature set “all features,” the MCC score follows the same pattern as the other metrics, i.e., exhibits higher values in the four-class setting. Strikingly, the performance boost achieved by reducing to four classes was especially high on “all features.” This can be explained by the fact that the eight-class version of the “all features” dataset contains four underrepresented classes that significantly degrade performance. This hypothesis is strengthened by the clinical observation, that the four cancer entities that are not included in the four-class setting (Kidney, Breast, Colorectal, ENT) do substantially differ from each other and the other four entities. This would mean that LOP prediction is clinically easier in our eight-class setting. Breast cancer is nearly exclusively seen in women and kidney cancer has a very different behavior. Therefore, the reduced performance in the eight-class setting will be mainly due to the mentioned class imbalance.

Regarding the ML methods used, we observed that the GBT method outperforms the RF. On one of the six datasets all measured performance values are higher for the GBT method (four-class “all features”). On the other five datasets, the majority of measured performance metrics is higher for the GBT method. This observation coincides with findings in ML research. These findings attribute a higher performance to the GBT method, in general, while the performance of the RF can be similarly high or even higher (27, 28).

Overall, we see that the LOP prediction performance on low-dimensional data is at the same level as the performance of related approaches using high-dimensional data (7–15). In our setting, this high performance (Accuracy: 93.6%, MCC: 0.917) was achieved with a GBT classifier on “all features,” i.e., on the dataset containing the TBS. Including the TBS significantly increased the prediction performance although it has not reached the top performance of high-dimensional approaches (9, 11–14). Their LOP predictors achieved Accuracy values of 95–97%. We assume that the performance of our low-dimensional approach can be optimized further. This optimization could be achieved by including other or additional routine clinical data. Moreover, other ML methods could be tested for their LOP prediction performance.

### 4.2 The predictive power of our feature sets

The LOP prediction performance on the feature sets “core features” and “core features and TM” was solid, but not remarkable. This is consistent with the conclusion from the previous paragraph that these feature sets are limited in their information content. This limitation reduces their predictive power in LOP prediction. By adding the TBS, i.e., the distribution pattern of metastases, to the dataset (“all features”), the LOP prediction performance increased significantly. Moreover, the TM and TBS received a large share of the overall FI when they were introduced to the dataset. As a consequence, the quartiles of the FI values decreased with increasing number of considered features. From these observations, we conclude that the TM and, in particular, the TBS add valuable information for LOP prediction to the dataset. This is a striking result considering that their limited availability makes the classification itself more difficult. Including the TM made the missing value imputation less stable due to the low availability of CEA and CA 19–9. Including the TBS reduced the dataset size significantly, because their extremely low availability required us to dispense with most cancer cases. Nevertheless, the TBS contributed to a remarkable increase in prediction performance. Furthermore, they led to a more balanced decision making which can be concluded from the reduced difference between Precision and Recall on “all features” compared to the other two feature sets. Reasoning on the predictive performance of individual features can be found in Supplementary material. A particularly striking observation was the decreased importance of the feature sex when not considering the LOP classes Breast, Colorectal, ENT, and Kidney anymore. This drop in FI for the sex could be due to the high number of female patients in the breast cancer group, while the ratio between men and women in the other entities is much more balanced.

### 4.3 The benefits of low-dimensional data for LOP prediction

When providing “all features” to the ML methods we achieved very high LOP prediction performance on low-dimensional data almost reaching the performance of high-dimensional approaches. Due to their slightly better performance, the high-dimensional approaches might appear more suitable for clinical LOP prediction. However, performance alone is not suitable for determining the quality of an LOP prediction system for clinical practice. This is because the performance only indicates how often the class predicted to be most probable was correct. Instead, it must be considered that the ML algorithm supports the oncologist in his decision; it does not make the decision for him. It could therefore also output LOP probabilities instead of the most probable class alone. Based on such a probabilistic overview, the oncologist could make their decision including their own prior knowledge. Thus, eventually the output of the LOP prediction system would enhance the oncologist’s knowledge in a data-driven manner instead of replacing it. The performance of the LOP prediction system alone cannot measure the quality of such a decision. We therefore believe that the small reduction in performance is justifiable; especially when one considers the clear advantages our low dimensional approach has in a practical setting. Our approach only needs routine clinical data, i.e., features readily available from a diverse patient population without specialized examinations. This restriction enables a cost-effective, user-friendly, and explainable LOP prediction for CUP patients which could be implemented by a clinical decision support system. The explainability is introduced by the chosen ML methods. While high-dimensional input data requires the application of artificial neural networks, which lack explainability, our low-dimensional approach allows the use of explainable tree-based methods like RF and GBT. Further decision support could be achieved by using probabilistic models such as Gaussian Process Models additionally to or instead of tree-based methods. Using such models would require some preprocessing of categorical variables but, on the other hand, add a statistically sound basis to the explainability of the LOP prediction. Moreover, as a future vision, our low-dimensional approach could enable a continuously learning LOP prediction system. Automated ETL processes could be used to update such a system with new patient data on an ongoing basis. These regular updates could improve the LOP prediction performance continuously. However, the data preparation process is currently still too complex and time-consuming for an automated ETL process (29). Overall, we consider the benefits of low-dimensional data for LOP prediction to outweigh the minor reductions in performance.

### 4.4 Limitations of our work

Our results show that low-dimensional data are well suited for LOP prediction, but our work has a few limitations beyond that. Firstly, our results do not reveal whether the performance improvements through adding the TM and TBS to the input data result specifically from these features. An alternative hypothesis is that the improved performance results merely from the ML method receiving more clinical information. Moreover, the significant performance gain through adding the TBS could be a result of a documentation bias. The radiologists knew the LOP when creating the RECIST evaluations of the cancer cases, based on which we created the TBS. The choice of documented target and non-target lesions might have been influenced by prior knowledge on the LOP. On the other hand, the RECIST guideline ensured the best possible objectivity. To improve the objectivity further, researchers could use different representations of the distribution of metastases. At UHC the documentation according to RECIST criteria was the only structured documentation representing the distribution of metastases.

Another limitation of our work is the restriction to eight rather broad LOP classes. Related works have considered more classes and sometimes even subclasses, which made their classification setting more difficult. Thus, for them, it was more difficult to achieve a high classification performance. We restricted to LOP classes that CUP patients have been assigned to post-mortem. So, we argue that many of the LOP classes considered by related work will not be relevant for deciding the treatment for CUP patients in practice. Additionally, some related works exceeded the capabilities of our approach by predicting the cancer subtype. Such an advanced prediction can further support treatment decisions. Moreover, some subtypes differ significantly in characteristics such as the distribution pattern of metastases. These significant differences may make differentiation of sybtypes easier than differentiation of higher-level cancer types. However, our results show that our RF- and GBT-based models can classify the different patterns that emerge in the subtypes into common cancer classes very well. Regarding the potential clinical disadvantage of not predicting the subtype, we argue that the subtype can be determined by entity-specific examinations once the LOP has been detected. What remains as a limitation is that we could not sufficiently test the feature set “all features” in the eight-class setting. When only considering four instead of eight classes, the FI of the feature sex dropped significantly. This clear reduction in FI shows that the eight-class classification task differs significantly from the four-class task. Due to the underrepresentation of the classes “Breast,” “Colorectal,” “ENT,” and “Kidney” in the eight-class version of the “all features” dataset, we did not obtain a reliable performance measurement of the LOP prediction based on the TBS in the eight-class setting. This limitation could be mitigated by repeating the experiments on a more balanced dataset. The class balance could be increased by including data from further cancer centers also documenting their study progress according to RECIST v1.1. Another step remaining as future work is the clinical or external validation of our results. Such a validation should include examining the effects of our data compilation decisions on the LOP prediction.

### 4.5 Conclusion and future work

All in all, the robust classification performance on all datasets serves as a proof-of-concept that LOP prediction on low-dimensional clinical information works well. We achieved remarkable classification performance in particular when the prediction models were given the distribution pattern of metastases. The low dimensionality of our prediction approach increases its practical applicability in data-driven LOP prediction significantly. Future work could now aim for optimizing the classification results by using more or different clinical routine data as input values. Additional optimization is possible by increasing the number of cancer cases in the datasets through collaboration with further clinics. This would address the issues of the small dataset sizes and the biases possibly introduced by including the TBS. Moreover, other ML methods such as probabilistic models as well as ensembles of ML algorithms could be tested for their LOP prediction performance on low-dimensional clinical information. Above all, however, it is key to investigate whether our approach delivers reliable LOP predictions for CUP patients. Externally or clinically validating the reliability of our low-dimensional LOP prediction approach is crucial before deploying it in clinical practice. With its focus on practical applicability, our approach could optimize the prognosis of CUP patients effectively.

## Data Availability

The data analyzed in this study is subject to the following licenses/restrictions: the applied data anonymization strategy was agreed with the Ethics Committee and the Data Protection Department of University Hospital Cologne on the condition that the data is only made available to employees of the clinic. Reasonable requests to access the analyzed datasets should be directed to corresponding author upon which they will be discussed with the Ethics Committee and the Data Protection Department. Requests to access these datasets should be directed to JG, julia.gehrmann1@uk-koeln.de.
