# Supplementary material for: Seeing the primary tumor because of all the trees: Cancer type prediction on low-dimensional data
Source: Front Med (Lausanne). 2024 Aug 27;11:1396459. doi: 10.3389/fmed.2024.1396459 (PMC11385615; doi:10.3389/fmed.2024.1396459)
Supplement: Supplementary file 1 [file Data_Sheet_1.docx]

*Supplementary Material*

# Details on Related Work

In recent years, several data-driven approaches predicting the Location of the primary tumor (LOP) have been proposed. All of them apply supervised classification, i.e. assigning LOP classes to patients (classification) with known LOPs (supervision) (1-9). This allows the detection of classification patterns specific to the well-defined LOP classes as well as performance estimation by comparing the actual LOPs with the predicted class. The resulting classification models can still be applied for LOP prediction in CUP cases because the LOP is the target variable and not needed as an input parameter for the system.

## Tree-based LOP prediction on genomic data

In 2020, Penson et al. proposed a RF classifier predicting the LOP based on the patient’s sex and genomic data covering discrete molecular alterations as well as mutational signatures. This data was obtained from the MSK-IMPACT study. Considering 22 LOP classes, they achieved an average Accuracy of 74% in 5-fold cross-validation (CV) (1). He et al. also investigated the LOP prediction performance of an RF classifier, but training it only on gene mutation data and considering only 13 LOP classes. Their data source was the International Cancer Genome Consortium (ICGC). With their approach they achieved an average Accuracy and F1-Score of 88% in 10-fold CV (2). Integrating information on gene expression to their input data and enlarging their dataset by additionally including data from The Cancer Genome Atlas (TCGA), they could increase the average Accuracy in 10-fold CV to 96% in internal testing and 90% in external validation even when distinguishing between 20 LOP classes (3). Instead of training a single RF on both gene expression and mutation data, Nguyen et al. created an ensemble of RFs on gene mutation data. Each RF solved the binary classification task of distinguishing one of the 35 classes against all others. With this approach they achieved an Accuracy of 91% in internal and 89% in external validation. Their dataset was compiled from the Hartwig Medical Foundation cohort and the Pan-Cancer Analysis of Whole Genomes (PCAWG) cohort (4). Another tree-based method was examined by Liu et al. who used the XGBoost approach to create three GBT classifiers predicting the LOP based on gene expression, DNA methylation and somatic mutation data, respectively. These classifiers achieved mean Accuracy scores of 95% (gene expression), 88% (DNA methylation) and 44% (somatic mutations) in 10-fold CV when distinguishing 21 LOP classes. The datasets were obtained from ICGC and TCGA (5). In 2021, Miao et al. created another GBT classifier with the XGBoost approach. It based the LOP prediction on expression quantitative trait loci downloaded from the Gene Expression Omnibus (GEO) and TCGA. Their approach achieved an average Accuracy of 96% in 10-fold CV (6).

## LOP prediction involving Deep Learning

Besides tree-based methods, DL has been examined regarding its performance in LOP prediction. Zhao et al. trained a CNN to discriminate 32 LOP classes based on the gene expression profile of the primary tumor tissue. The DL approach achieved an Accuracy score of 97% in internal and 93% in external validation. Their data sources were the Broad Institute's Genome Data Analysis Centre and TCGA (7). Lu et al. applied a CNN as well but trained it on pathological WSI and the patient’s sex. Thus, they used images instead of genomic data as the main information source for LOP classification which resulted in an Accuracy of 83% in internal and 80% in external validation when distinguishing 18 LOP classes. Some of the images used were obtained from TCGA and the CPTAC Pathology Data Portal and some were curated in-house (8). Vibert et al. did not use DL for the classification but for dimensionality reduction. They applied an Variational Autoencoder to obtain a 100-dimensional representation of the full gene expression profile and then assigned the encodings to one of 39 LOP classes using a RF and a *k*-Nearest Neighbor classifier. Both classifiers achieved an average Accuracy score of 96% in 3-fold CV. The data sources for this project were the SHIVA 01 clinical trial at Curie Institute, TCGA, the Genotype-Tissue Expression (GTEx) project, and the Human Protein Atlas (9).

## Current trends in LOP prediction

All in all, we see a clear trend towards LOP prediction using tree-based methods on genomic data such as gene expression, gene mutation or DNA methylation data. The majority of all presented approaches uses input data that is not created in clinical practice by default and thus introduce additional costs. Most of the previous work on LOP prediction obtained the data used from already curated cohorts, from previous studies, or from registries like TCGA, ICGC, GEO, or CPTAC. Only Lu et al. curated some of the input data themselves. Moreover, some of these approaches use data that is not available in CUP cases, such as genomic data of the tissue of the primary tumor. To our knowledge, it has not yet been investigated whether routine clinical features are sufficient as input data for a well-functioning LOP classification of cancer cases. Utilizing such input data would lower the cost of LOP prediction for CUP patients and would enable its clinical deployment.

# Curation of datasets

## Data sources

At the start of our study the most comprehensive collection of structured information on cancer patients at UHC could be found at the Clinical Cancer Registry (CCR). Since 2016, the CCR team is obliged by law to report cancer cases to the Cancer Registry of the federal state in a structured form according to the ADT/GEKID core dataset (10). Before 2016, the German Cancer Society determined how cancer cases are reported in the CCR. We used the database of the CCR to identify suitable cancer cases according to our inclusion criteria and exported those.

Additionally, we obtained blood test results for the identified cancer cases from the Clinical Information System (CIS) through the Medical Data Integration Center (MeDIC) Cologne. The CIS contains all clinical information gathered at UHC which are relevant for diagnosis and treatment decisions. The MeDIC Cologne is the Data Integration Center instance at UHC. Like other Data Integration Centers at German University Hospitals it was established in the course of the Medical Informatics Initiative (MI-I) in Germany to foster interoperability, harmonization and standardization of clinical RWD for research (11).

A third data source was the Radiological Study System (RSS). It stores key images for cancer cases which are part of a study as well as the course of the disease in form of structured evaluations following RECIST v1.1 criteria (12).

Choosing the CCR, the CIS and the RSS as the data sources for our study, provided us with the most complete dataset of individual cancer cases that was available at UHC without further documentation and structuring effort.

## Data engineering and integration

We could not use the raw data from the clinical systems at UHC immediately but needed to perform some steps of data engineering to preprocess the data sufficiently for a meaningful analysis. These steps included case and feature selection prior to actual data access, data transformation, value selection, dataset creation, missing value imputation and anonymization. If not indicated otherwise all steps were implemented and executed in python 3.7.9. The following python libraries were used: *numpy* v1.21.5, *pandas* v1.3.5, *matplotlib* v3.5.3 and *seaborn* v0.11.2. Some of the methodological decisions described in the following are based on results outlined in section D.1 “Characteristics of the compiled dataset”.

### Case selection

We aimed at compiling a dataset that is comprehensive enough to train a well-working LOP prediction system. Thus, we chose to include cancer cases of a rather long period. Specifically, we considered cancer cases documented in the CCR that were diagnosed at UHC between 01.01.2000 and 30.06.2021.

Thereby, we only included cancer cases with known LOP, i.e. no CUP cases. This decision enabled us to create supervised classifiers which are trained on cancer cases with known classes. Additionally, we only included primary, i.e. non-recurrent, cancer cases, and the patient was required to be an adult, i.e. at least 18 years old at the date of diagnosis. These criteria ensure sufficient homogeneity of the dataset. In accordance with best practice for clinical trials, we excluded cases involving a patient who had another cancer diagnosis within 5 years prior to or after the diagnosis in our dataset.

The vast majority of primary tumors found in autopsies of CUP patients are located in the Lung, Pancreas, Kidney, Liver, Genitals, Colorectal area, Ears-Nose-Throat (ENT) or the Upper Gastrointestinal tract (Upper GI) (13-15). Therefore, we only considered cases of the following cancer entities: Lung, Pancreas, Kidney, Liver, Breast, Colorectal, ENT, and Upper GI. Genital cancer cases were unfortunately not documented in the component of the CCR we had access to.

### Feature selection

When selecting the features for LOP prediction we leveraged our team’s medical

expertise and followed the principle of data minimization: as little as possible as much as necessary. From the data available in the ADT/GEKID core dataset we chose to include the age, the sex and histological specifications (the grading, N, L and V value) as input features. The diagnosed cancer type was exported to serve as the target feature. From the data available in the CIS we selected the blood test results for the analytes leukocytes, C-reactive protein (CRP), Hemoglobin (HB) and the two tumor markers (TM) Carbohydrate Antigen 19-9 (CA 19-9) and Carcinoembryonic Antigen (CEA). We chose these analytes because they are regularly measured for cancer patients in clinical practice and therefore widely available across cancer entities.

Moreover, we considered the distribution pattern of each patient’s metastases as a high-quality predictor of the LOP. The exact number of metastases per organ was not documented in a structured form at UHC but we could approximate these numbers by defining an organ-specific Tumor Burden Score (TBS) and calculating it based on the structured RECIST evaluations documented in the RSS. The specification of the TBS can be found in Supplementary Table 1.

### Data access

The age, sex, histological specification and ICD10-codes of suitable cancer cases were obtained by sending an SQL query to the CCR database which specified our inclusion and exclusion criteria for case selection. Additionally, our SQL query requested the prefix of the T value, the date of each histological examination, the Patient ID (PID), the CCR case ID and the diagnosis date for the exported cases which enabled value selection in and integration of the individual exports down the line. PIDs were extracted from the CCR export and used to obtain the remaining clinical data from the CIS and the RSS. These two systems use the CIS case IDs instead of CCR case IDs. So, we needed the PIDs for record linkage. The record linkage procedure is explained in section B.2.5 Value Selection. The blood test results were exported from the CIS using an SQL query requesting the chosen analytes for the exported PIDs. Besides the test values, we exported the examination date, the unit and some flags specifying the actuality of the respective value. This additional information was needed for data preprocessing and case-specific choice of value. Eventually, we accessed the RSS through its user interface. We provided the extracted PIDs to the system and determined the overlap of these with the study participants in the RSS. For the study participants we exported the names of studies they participated in, the respective baseline assessment dates as well as the location of all target and non-target lesions according to RECIST v1.1 criteria (12).

### Transformation

Almost all exported data could be used without further modifications. The only exceptions were the ICD10-codes, the histological specifications, the RSS export as a whole and the leukocytes values from the CIS export.

The ICD10-codes were translated to the cancer type they represent, i.e. to Breast, Colorectal, ENT, Kidney, Liver, Lung, Pancreas, or Upper GI. The histological specifications partly had suffixes specifying subcategories. These suffixes have been removed such that we only considered grading values of 0 to 4, N values of 0 to 3, V values of 0 to 2 and L values of 0 or 1 without any subcategories.

For each study participant, the RSS data provided the location for a set of documented target and non-target lesions according to RECIST v1.1 criteria (12). Based on this information, we calculated organ-specific TBS as specified in Supplementary Table 1 for 19 organ types (Lymphnodes, Kidney, Adrenal Gland, Bladder, Heart, Oesophagus, Stomach, Pancreas, Liver, Omentum, Bones, Lung, Skin, Spleen, Brain, Mamma, Intestine, Thyroid Gland, Other Organs).

Additionally, we needed to harmonize the units of leukocytes values in the CIS export. They were given either in cells per microliter or 1.000.000.000 cells per liter. To obtain comparable values we converted the values given in cells per microliter to 1.000.000.000 cells per liter by dividing them by 1000.

All other exported features were usable without simplification, meta-feature calculation and unit harmonization. Normalization and scaling of feature values was not necessary for the tree-based ML approaches we used for LOP prediction (4).

| **Number of target lesions** | **Number of non-target lesions** | **Description** | **TBS value** |
| --- | --- | --- | --- |
| 0 | 0 | No metastases | 0 |
| 0 | ≥ 1 | Only small metastases | 1 |
| 1 | 0 | One measurable metastasis | 2 |
| 2 | 0 | Two measurable metastases | 3 |
| ≥ 1 | ≥ 1 | At least two metastases | 4 |

**Supplementary Table 1**: Specification of organ-specific Tumor Burden Score (TBS) assignment to study participants based on the number of target and non-target lesions documented for the respective patient in the respective organ.

### Value selection

For the majority of cancer cases the database exports contained more than one histological examination and blood test result because the patients underwent multiple examinations throughout diagnosis and treatment. Some patients even participated in several studies contained by the RSS so that we additionally had more than one set of TBS for these patients.

In cases for which more than one histological examination result was documented in the CCR, we select the histological examination based on the prefix of the T value and the examination date. If there were one or more results with T prefix “p” (i.e. a pathological examination result) that were examined not more than 3 months after the first result with T prefix “c” (i.e. a clinical examination result) we took the earliest of those. If there was no result with T prefix “p” or all results with T prefix “p” were examined more than 3 months after the first result with T prefix “c” we chose the overall earliest result. To minimize the missingness rates we filled missing values for the histological specifications (the grading, N, L and V value) with the maximal value of the respective feature over all histological examination results available for the respective case.

By matching PIDs from the CCR export to PIDs in the CIS export we could identify cancer cases for which blood test results are available. For these combinations of cancer cases and blood test results sharing the same PID, we extracted the CCR case ID, the diagnosis date, the date of the blood test, the analyte, the value and flags specifying the actuality of the result. For each chosen blood test analyte, we selected the result of the examination with minimal temporal distance to the diagnosis. However, for some examinations we found several non-identical values in the CIS export which can happen if values are corrected after their first entry to the system. We selected the most current value which was identifiable by associated flags specifying the actuality of the respective value in the export. If an examination had several values with identical flags we took the mean of these values in case no pairwise difference between two of these values was too large. If any of the pairwise distances was too large, we excluded the respective examination completely to avoid ambiguity. We considered the pairwise distance as too large if it was larger than 10% of the span of the normal values for the respective analyte.

Furthermore, we matched the PIDs from the CCR export to the PIDs in the transformed RSS export so that we could identify cancer cases for which the distribution pattern of metastases might be available. For these combinations of cancer cases and study participations sharing the same PID, we extracted the CCR case ID, the cancer type, the diagnosis date, the baseline assessment date of the study and the organ-specific TBS calculated before.

For each study contained in the RSS export we created a list of cancer entities considered in the respective study. We removed all combinations of cancer cases and study participations from our dataset in which the cancer type of the case and the cancer entities considered by the study did not match. Additionally, we removed all combinations of cancer cases and study participations in which the baseline assessment date of the study took place more than 3 months before or more than 10 years after the diagnosis. For cases that were still associated with several eligible studies, we assigned the maximal organ-specific TBS score over all such studies for each considered organ.

### Data quality check

We used visualizations to monitor basic data quality throughout the data preprocessing process. On the one hand, these visualizations enabled us to make informed decisions based on the overview they provide. On the other hand, it enabled us to ensure that we did not introduce inaccuracies or biases throughout preprocessing. The data Accuracy was monitored by visualizing the feature distributions and checking their plausibility. The distribution of numerical features was visualized with a histogram accompanied by a respective kernel density estimation (kde) plot using the function *histplot* from the python library *seaborn* setting the parameter *kde* to TRUE. The distribution of categorical variables was visualized with a barplot using the *seaborn* function *countplot*. Moreover, we monitored the completeness by visualizing the missingness rates of individual features as a barplot using the *seaborn* function *barplot*.

### Dataset compilation

The transformation and value selection process was executed on the three exports (CCR, CIS, RSS) individually but already integrated the CCR case IDs in all of them. Thus, we could merge the individual exports on the CCR case ID. However, we saw high missingness rates for some features in the visualization of data completeness. So, we decided to create three datasets and perform the remaining preprocessing steps individually for these datasets. The first one, the “core features” dataset, solely contains features exhibiting missingness rates below 35%. These are the sex, the age, the histological specifications (the grading, N, L and V value), and the blood test analytes leukocytes, CRP and HB. The second dataset, the “core features and TM” dataset, contains the same features as the “core features” dataset plus the two TM CA 19-9 and CEA. We created these minimally different datasets because CA 19-9 and CEA exhibit much higher missingness rates (77% and 68%, respectively). So, we expected more robust value imputation for the “core features” dataset compared to the “core features and TM” dataset. The third dataset, the “all features” dataset, contains all selected features but only those cancer cases for which organ-specific TBS could be calculated. We made this decision because the TBS scores had a missingness rate of 97.6%. So, a reliable missing value imputation was not feasible for them.

Furthermore, we noticed that the LOP classes Kidney, Breast, Colorectal, and ENT are underrepresented in the “all features” dataset with a prevalence of 14, 10, 8 and 5, respectively. As such small class sizes significantly affect the performance of ML methods and restrict the applicability of 10-fold CV, we decided to create another version of each of the three datasets only containing cases belonging to the remaining four LOP classes (Lung, Upper GI, Pancreas, Liver). The compilation of the total of six datasets is shown in the main text in Figure 1.

### Missing value imputation

There are two approaches for dealing with missing values in a dataset. The first is removing incomplete samples from the dataset. The second is imputing missing data values (16). Aiming for a maximal dataset size we decided to impute missing values. The most widely used approach for value imputation is mean and mode imputation for numerical and categorical features, respectively (16). This, however, tends to artificially introduce biases and decrease the variance in the dataset (17). Therefore, we used Multiple Imputation by Chained Equations (MICE) instead. It is shown to be a robust method for accurately imputing numerical as well as categorical values even in small datasets (17, 18).

For implementing MICE, we applied the function mice from R package mice and set the number of multiple imputations “m” to 10, the number of iterations “maxit” to 10, “writeLines” to FALSE and the method for single imputations “method” to "rf" for Random Forest. The information about the LOP class of individual patients was not provided to the imputation algorithm to avoid introducing a bias to the imputed values.

### Anonymization

At first, all selected input features were analyzed regarding their risk of re-identification. The only features that we had to delete completely in the course of anonymization are those we needed throughout data preprocessing and integration (PID, case IDs, dates). According to EMA Policy 007 Implementation Guideline, the remaining features, i.e. the chosen input features are considered non-identifying (19). Quasi-identifying features were determined following the approach of Jakob et al. based on the framework of Malin et al. (20, 21). Using a questionnaire six team members (management, medical domain experts, technical experts) assessed the remaining features in terms of the three aspects of replicability, resource availability and distinguishability of different feature expressions. For this, they assigned scores from 1 (low) to 3 (high) to every combination of feature and aspect. The average score over all six survey participants was calculated for every combination of feature and aspect. The three aspect-specific scores per feature were summed up to a final re-identification score. Features having a score higher than 5 are considered quasi-identifying. We generalized these quasi-identifying features such that *k*-Anonymity with *k* set to 5 was established. Value groups that were created in the course of generalization were replaced by their means to get numerical values again. For this we applied the comprehensive and widely used anonymization tool ARX (22), which is recommended and used by the TMF. ARX identifies all possible anonymization solutions for the provided dataset and the settings of the user. For all solutions it displays a residual risk score and a usability score. The user can then choose the anonymization solution that should be applied (23). We optimized the anonymization for usability which was sufficient for internal exchange of data within UHC. The upper limit for the residual risk score for any kind of attack supported by ARX (Prosecutor, Journalist, Marketer) was set to 0.1. Additionally, we allowed ARX to suppress up to 5% of the patients to optimize the anonymization.

# Details on applied LOP prediction methods and their implementation

For our LOP prediction system we chose a supervised classification approach, i.e. we trained mathematical models to assign cancer cases to cancer types (classification) by providing the models with example cases (samples) whose cancer type is known (supervision). The hyperparameters of the mathematical models were optimized and the performance of our prediction systems was evaluated comprehensively. Additionally, we estimated the importance of individual input features for the classification to obtain some understanding of the decision process happening in the models. All these steps were implemented by use of the python library *sklearn* v1.0.2 (24).

## Classification

As a basis, both applied ML methods use the Decision Tree (DT) approach. It is a method for supervised classification that splits the training dataset repeatedly by the feature which respectively maximizes the decrease in entropy of the class variable. To assess the entropy different measures like the Gini-Index or Cross-Entropy can be applied (25, 26). However, single DTs suffer from high variance and tend to overfit the training data. To solve this issue the RF approach has been developed. It trains different DTs each on a different subset of the available features resulting in de-correlated DTs. The variance between the DTs can additionally be increased by bootstrapping, i.e. not only considering a random subset of features when training the individual DTs but also a random subset of samples. The de-correlated DTs then democratically decide on a class for a given sample. The approach exhibits significantly lower variance and better generalization performance than the single DTs (25, 27). The GBT approach trains several DTs as well but sequentially. Thereby, the first DT determines a classification probability for each considered class. The following trees in the sequence each improve the prediction of the samples that were misclassified before. This is achieved by aiming for a minimization of the residual loss left over by the DT sequence build so far. Weighing its prediction by a learning rate *γ* the new tree is added to the tree sequence (25, 28).

We implemented the approaches using the *sklearn.ensemble* functions *RandomForestClassifier* and *GradientBoostingClassifier*, respectively*.*

## Classification evaluation and hyperparameter optimization

The Precision, the Recall, and the F1-Score are only defined for binary classifications, i.e. classifications only considering two classes. These metrics (number of true positives, false negatives, true negatives and false positives) were firstly determined for each class individually (considering the classification “this class” vs. “any other class”) and then aggregated by macro-averaging, i.e. determining the averages of the class-specific metrics.

Furthermore, the performance was evaluated on a part of the compiled dataset that was not seen during the model training procedure, i.e. on a left-out test dataset. To reduce the influence of the choice of cancer cases in the test dataset we performed 10-fold CV. This means that we divided the compiled dataset in ten equally sized parts and trained each model ten times, each time leaving out one of the ten parts during model training. The left-out part was used as the test dataset. To additionally tune the performance of the models we performed hyperparameter optimization by including a second (inner) 10-fold CV inside the first (outer) CV. This approach is known as nested cross-validation (NCV). The inner CV determined an optimal parameter set from a pre-defined list of parameters and eligible values. For this, it applied a grid search aiming to find the optimal set of parameters for the respective model trained on the current training dataset of the outer CV.

The determined optimal set of parameters was used to train and evaluate the model in the outer CV. The division of the cancer cases into a training and a test dataset was done in a stratified manner, both in the outer and in the inner CV. This mean that the distribution of class labels in the training and the test dataset corresponds to the distribution in the original dataset.

For evaluation and hyperparameter optimization we solely applied the library *sklearn*. The calculation of the performance metrics was implemented using the functions *Accuracy_score, f1_score, Precision_score, Recall_score* and *matthews_corrcoef* of the module *metrics.* The confusion matrix needed for micro-averaging the binary classification metrics was obtained through the function *confusion_matrix* which is also provided by the module *metrics.* For the NCV we used the functions *StratifiedKFold* (creating splits for inner and outer CV) and *GridSearchCV* (performing the grid search) from module *model_selection*.

## Feature importance estimation

To understand the relevance of the individual input features for LOP prediction, we determined their percentual importance in the implemented classification task. When using tree-based methods the importance of an individual input feature is given by the average of the decrease in class entropy over all splits in which it has been used as the separating feature (28, 29). When training a RF or GBT classifier with the library *sklearn* these percentual feature importance (FI) values are available for each feature in the attribute *feature_importances_* of the classifier object. We accessed the FI values through that attribute and visualized them in a barplot for each classification run using the *seaborn* function *barplot*.

Furthermore, we determined the first, second and third quartile of the determined FI values for each combination of dataset and applied ML algorithm. This enabled us to assign the features to four groups depending on their FI. Features whose FI does not exceed the first quartile are considered “low importance” features. With a FI above the first quartile and not more than the second quartile we classified a feature to have “medium low importance”. If a feature’s FI exceeds the second quartile but not the third we assign it a “medium high importance”. Features with a FI value above the third quartile are considered to have high importance. This grouping enables easier comparison of the FI across classification settings, i.e. combinations of datasets and ML algorithms.

# LOP Prediction Results in Numbers

## Achieved Performance Scores

| **Approach** | **Accuracy** | **Precision** | **Recall** | **F1-score** | **MCC** |
| --- | --- | --- | --- | --- | --- |
| RF on core features | 0.558 (0.006) | 0.415 (0.038) | 0.343 (0.009) | 0.341 (0.012) | 0.423 (0.008) |
| GBT on core features | 0.562 (0.007) | 0.407 (0.022) | 0.358 (0.012) | 0.363 (0.015) | 0.430 (0.010) |
| RF on core features and TM | 0.563 (0.007) | 0.447 (0.042) | 0.352 (0.010) | 0.351 (0.014) | 0.429 (0.010) |
| GBT on core features and TM | 0.570 (0.012) | 0.436 (0.026) | 0.379 (0.015) | 0.388 (0.018) | 0.441 (0.016) |
| RF on all features | 0.845 (0.046) | 0.587 (0.114) | 0.600 (0.105) | 0.586 (0.110) | 0.808 (0.057) |
| GBT on all features | 0.841 (0.047) | 0.627 (0.127) | 0.637 (0.125) | 0.625 (0.125) | 0.802 (0.059) |

**Supplementary Table 2:** Performance of the two applied machine learning methods Random Forest (RF) and Gradient Boosted Trees (GBT) on the three feature sets “core features”, “core features and TM” and “all features” in the 8-class classification task of assigning cancer cases to one of eight classes (Breast, Colorectal, ENT, Kidney, Liver, Lung, Pancreas, Upper GI) each representing a different location of the primary tumor (LOP). The table shows the mean performance values across ten nested Cross-Validation (NCV) iterations in terms of Accuracy, Precision (macro-averaged), Recall (macro-averaged), F1-score and Matthews Correlation Coefficient (MCC). In brackets we provide the standard deviations of the performance values across the ten NCV iterations.

| **Approach** | **Accuracy** | **Precision** | **Recall** | **F1-score** | **MCC** |
| --- | --- | --- | --- | --- | --- |
| RF on core features | 0.572 (0.018) | 0.527 (0.042) | 0.442 (0.022) | 0.450 (0.026) | 0.344 (0.03) |
| GBT on core features | 0.574 (0.012) | 0.525 (0.039) | 0.455 (0.011) | 0.467 (0.015) | 0.350 (0.019) |
| RF on core features and TM | 0.574 (0.033) | 0.561 (0.081) | 0.450 (0.034) | 0.455 (0.041) | 0.348 (0.051) |
| GBT on core features and TM | 0.591 (0.026) | 0.548 (0.050) | 0.492 (0.035) | 0.504 (0.041) | 0.382 (0.041) |
| RF on all features | 0.913 (0.039) | 0.923 (0.034) | 0.911 (0.042) | 0.912 (0.038) | 0.884 (0.053) |
| GBT on all features | 0.936 (0.025) | 0.947 (0.021) | 0.934 (0.023) | 0.935 (0.023) | 0.917 (0.032) |

**Supplementary Table 3**: Performance of the two applied machine learning methods Random Forest (RF) and Gradient Boosted Trees (GBT) on the three feature sets “core features”, “core features and TM” and “all features” in the four-class classification task of assigning cancer cases to one of four classes (Lung, Upper GI, Pancreas, Liver) each representing a different location of the primary tumor (LOP). The table shows the mean performance values across ten nested Cross-Validation (NCV) iterations in terms of Accuracy, Precision (macro-averaged), Recall (macro-averaged), F1-score and Matthews Correlation Coefficient (MCC). In brackets we provide the standard deviations of the performance values across the ten NCV iterations.

## Chosen Hyperparameter Combinations

| **Parameter Set (number of DTs, max tree depth, entropy measure, bootstrapping?)** | **# chosen in “core features”**  **(8 classes)** | **# chosen in “core features and TM”**  **(8 classes)** | **# chosen in “all features”**  **(8 classes)** | **# chosen in “core features” (4 classes)** | **# chosen in “core features and TM”**  **(4 classes)** | **# chosen in “all features”**  **(4 classes)** | **# chosen in total** |
| --- | --- | --- | --- | --- | --- | --- | --- |
| (35, 10, cross, yes) | 0 | 0 | 0 | 1 | 0 | 0 | 1 |
| (50, 10, cross, yes) | 0 | 0 | 1 | 2 | 2 | 0 | 5 |
| (20, 5, gini, yes) | 0 | 0 | 0 | 0 | 0 | 1 | 1 |
| (35, 7, gini, yes) | 0 | 0 | 0 | 0 | 0 | 1 | 1 |
| (35, 10, gini, yes) | 0 | 0 | 0 | 2 | 1 | 0 | 3 |
| (50, 7, gini, yes) | 0 | 0 | 0 | 0 | 0 | 1 | 1 |
| (50, 10, gini, yes) | 3 | 1 | 2 | 0 | 2 | 1 | 9 |
| (10, 7, cross, no) | 0 | 0 | 0 | 0 | 0 | 1 | 1 |
| (20, 10, cross, no) | 0 | 0 | 1 | 1 | 1 | 0 | 3 |
| (35, 10, cross, no) | 0 | 0 | 0 | 3 | 0 | 2 | 5 |
| (50, 7, cross, no) | 0 | 0 | 0 | 0 | 1 | 0 | 1 |
| (50, 10, cross, no) | 1 | 0 | 0 | 1 | 2 | 0 | 4 |
| (20, 7, gini, no) | 0 | 0 | 1 | 0 | 0 | 0 | 1 |
| (35, 7, gini, no) | 0 | 0 | 1 | 0 | 0 | 1 | 2 |
| (20, 10, gini, no) | 2 | 0 | 2 | 0 | 0 | 1 | 5 |
| (35, 10, gini, no) | 1 | 5 | 2 | 0 | 0 | 0 | 8 |
| (50, 10, gini, no) | 3 | 4 | 0 | 0 | 1 | 1 | 9 |

**Supplementary Table 4**: Combinations of optimal hyperparameters for the Random Forest (RF) algorithm together with the number of nested Cross-Validation (NCV) iterations in which they were selected as optimal for the respective dataset/ in total. Combinations of RF hyperparameters that have not been chosen as optimal in at least one NCV iteration on at least one of the six datasets are not shown in the table.

| **Parameter Set (learning rate, max tree depth)** | **# chosen in “core features”**  **(8 classes)** | **# chosen in “core features and TM”**  **(8 classes)** | **# chosen in “all features”**  **(8 classes)** | **# chosen in “core features” (4 classes)** | **# chosen in “core features and TM”**  **(4 classes)** | **# chosen in “all features”**  **(4 classes)** | **# chosen in total** |
| --- | --- | --- | --- | --- | --- | --- | --- |
| (0.1, 3) | 10 | 10 | 2 | 10 | 10 | 2 | 44 |
| (0.1, 5) | 0 | 0 | 1 | 0 | 0 | 2 | 3 |
| (0.2, 3) | 0 | 0 | 3 | 0 | 0 | 1 | 4 |
| (0.2, 5) | 0 | 0 | 2 | 0 | 0 | 2 | 4 |
| (0.5, 3) | 0 | 0 | 2 | 0 | 0 | 2 | 4 |
| (0.5, 5) | 0 | 0 | 0 | 0 | 0 | 1 | 1 |

**Supplementary Table 5**: Combinations of optimal hyperparameters for the Gradient Boosted Trees (GBT) algorithm together with the number of nested Cross-Validation (NCV) iterations in which they were selected as optimal for the respective dataset/ in total. Combinations of GBT hyperparameters that have not been chosen as optimal in at least one NCV iteration on at least one of the six datasets are not shown in the table.

# Reasoning on Predictive Performance of Individual Features

For each clinical feature in our datasets, we obtained the importance of this feature for LOP prediction in the individual classification settings, i.e. its Feature Importance (FI). The classification setting is determined and influenced by all respectively considered features. Hence, the FI of the individual features must always be considered in the context of the other features also being available to distinguish the classes. Based on the quartiles of the FI values in a given classification setting, we assigned the features to groups of low, medium low, medium high and high importance for the respective classification. The FI quartiles decreased with an increase in the number of input features. This strengthens our finding that the TM and TBS which are added to the “core features” dataset in the datasets “core features and TM” and “all features”, provide useful information to the ML methods. The reason for this is that they get a significant share of the overall FI when added to the input data. Sharing the overall FI across more features naturally decreases FI quartiles.

The rather high FI of the TM CA 19-9 and CEA coincides with the current CUP diagnosis guideline of the European Society for Medical Oncology (ESMO). It lists both TM as useful biomarkers to identify the LOP (30). Moreover, CA 19-9 has been reported as a leading TM for pancreas and biliary carcinomas (31,32,33) with particularly high sensitivity and specificity for pancreas carcinomas (34). Besides that, an elevated CA 19-9 value appears as a potential TM for further considered cancer entities such as upper GI, lung, colorectal, and kidney (32). CEA is used as a leading tumor marker for biliary cancer (33,35), and colorectal cancer (36,37). In particular, CEA is the strongest TM in detecting and measuring the prognoses of colorectal carcinomas (38). Furthermore, it has previously been identified as a promising TM for Non-Small Cell Lung Cancer (NSCLC) (39). Despite being elevated in several cancer entities, both CA 19-9 and CEA exhibited rather high FI in all classifications in which they were provided. This indicates that their value adds important information for the LOP prediction and suggests that their elevation level differs in different cancer entities, just as different elevation levels of CA 19-9 result in varied diagnostic, staging, prognosis, and recurrence (32). In the eight-class setting their importance is particularly high in combination with the TBS, which suggests that the TBS and TM together are very well suited as predictors of the LOP class. On the other hand, the fact that they are non-entity specific explains why they were not assigned to the group of high importance features in all classifications.

When the TBS are provided, the group of highly important features mainly consists of TBS features. The TBS for the Lymphnodes, the Oesophagus, the Pancreas, the Lung and the Liver appear to be particularly important, as they belong to the high importance features in all 4 classifications in which they were known. The only non-TBS features assigned to this group in any classification on an “all features” dataset are the N value, the CA 19-9 value, the CRP value, and the age. This indicates the high predictive value of these features in combination with the distribution pattern of metastases. Furthermore, we observed a decrease of importance for many features when the TBS are provided. We therefore conclude that the distribution of metastases is of significant importance for LOP prediction. We would expect further improvement of classification performance if more fine-grained information on the distribution pattern of metastases would be available, e.g. the exact number of metastases per organ. Moreover, we would expect a more reliable and robust LOP prediction if the “all features” dataset contained more cases on which the ML methods could identify the patterns. Both the increased information depth and the larger dataset size can only be achieved with a change in documentation because the information about the exact number of metastases per organ is currently not captured in a structured manner at UHC and even the RECIST criteria are not evaluated for patients other than study participants. This results in a rather small number of cases in the “all features” dataset and in this sub-cohort, some organs show little or no metastases. Out of 336 cases for which the RECIST evaluations were available, the TBS for Heart, Omentum, Skin, Spleen, Mamma, and Thyroid Gland were 0 for at least 332 cases each. The TBS for Omentum, Skin, and Mamma were even 0 for every case. This explains why all classifications have assigned an FI of 0 to these features. A feature that is the same for all cases in the dataset cannot be used to differentiate the cases.

Besides the TM and TBS, some “core features” exhibited high FI values. For instance, the CRP value generally shows a very high importance. CRP is the only Acute-Phase Reactant Protein (APRP) we included in our dataset (40). APRPs are proteins whose production is increased in the course of an immune response to tumor growth. The elevation level of CRP differs in different cancer entities which explains its high predictive value (40,41). Furthermore, we observed high FI values for the features leukocytes and HB which is consistent with their mention as relevant factors in CUP diagnosis in the aforementioned ESMO guideline (29). In contrast, the finding that the age of a patient has a high importance in many classification settings was a surprise. A comparison with the database of the Centre for Cancer Registry Data has shown that the cancer entities included do not differ significantly in terms of the most common age at first diagnosis. The still high FI of age is possibly a result of random effects in the compilation of the datasets or an effect resulting from the combined consideration of age with other features.

The sex shows overall high FI in 8-class classification task. The reason for this is that “Breast” is by far the most prevalent class when considering all 8 LOPs (c.f. Supplementary Figure 4) and breast cancer is mostly diagnosed in women. Only 1% of cancer diagnoses affect men (42,43). When not considering “Breast” as a class anymore in the 4-class setting, the FI of sex drops because both sexes are more or less equally represented in the remaining LOP classes Liver, Lung, Pancreas and Upper GI. Only the GBT-based classification on the 4-class “core features” dataset still assigns a medium high importance to the feature sex. We explain this by the fact that the sex still adds useful information for distinguishing the LOP classes when information is limited, i.e. without the presence of TM and TBS. If the TM or TBS are provided to the ML method in the 4-class setting, the sex is of low to medium low importance without exception. This indicates that the TM and TBS features contribute more useful information than the sex if we distinguish LOP classes Liver, Lung, Pancreas and Upper GI.

Further observations regarding predictive performances of individual features might hint to previously unknown relationships between these features in certain cancer entities. Analyzing them and examining possible consequences or reasons remains as future work. It could investigate the actual importance and predictive performance of the individual features and of further suitable features that were not part of our datasets.

# Supplementary References

1. Penson A, Camacho N, Zheng Y, et al. Development of Genome-Derived Tumor Type Prediction to Inform Clinical Cancer Care. *JAMA Oncol*. 2020;6(1):84-91. doi:10.1001/jamaoncol.2019.3985

2. He B, Dai C, Lang J, Bing P, Tian G, Wang B, Yang J. A machine learning framework to trace tumor tissue-of-origin of 13 types of cancer based on DNA somatic mutation. *Biochim Biophys Acta Mol Basis Dis.* 2020 Nov 1;1866(11):165916. doi: 10.1016/j.bbadis.2020.165916.

3. He B, Lang J, Wang B, Liu X, Lu Q, He J, Gao W, Bing P, Tian G, Yang J. TOOme: A Novel Computational Framework to Infer Cancer Tissue-of-Origin by Integrating Both Gene Mutation and Expression. *Front Bioeng Biotechnol.* 2020 May 19;8:394. doi: 10.3389/fbioe.2020.00394

4. Nguyen L, Van Hoeck A, Cuppen E. Machine learning-based tissue of origin classification for cancer of unknown primary diagnostics using genome-wide mutation features. *Nat Commun*. 2022;13(1):4013. doi:10.1038/s41467-022-31666-w

5. Liu H, Qiu C, Wang B, Bing P, Tian G, Zhang X, Ma J, He B, Yang J. Evaluating DNA Methylation, Gene Expression, Somatic Mutation, and Their Combinations in Inferring Tumor Tissue-of-Origin. *Front Cell Dev Biol.* 2021 May 3;9:619330. doi: 10.3389/fcell.2021.619330.

6. Miao Y, Zhang X, Chen S, Zhou W, Xu D, Shi X, Li J, Tu J, Yuan X, Lv K, Tian G. Identifying cancer tissue-of-origin by a novel machine learning method based on expression quantitative trait loci. *Front Oncol.* 2022 Aug 9;12:946552. doi: 10.3389/fonc.2022.946552.

7. Zhao Y, Pan Z, Namburi S, et al. CUP-AI-Dx: A tool for inferring cancer tissue of origin and molecular subtype using RNA gene-expression data and artificial intelligence. *EBioMedicine*. 2020;61:103030. doi:10.1016/j.ebiom.2020.103030

8. Lu MY, Chen TY, Williamson DFK, et al. AI-based pathology predicts origins for cancers of unknown primary. *Nature*. 2021;594(7861):106-110. doi:10.1038/s41586-021-03512-4

9. Vibert J, Pierron G, Benoist C, et al. Identification of Tissue of Origin and Guided Therapeutic Applications in Cancers of Unknown Primary Using Deep Learning and RNA Sequencing (TransCUPtomics). *J Mol Diagn*. 2021;23(10):1380-1392. doi:10.1016/j.jmoldx.2021.07.009

10. Dudeck J, Wächter W, Altmann U, Katz F. The definition of a new uniform basic data set for hospital cancer registries in Germany. *MIE Proceedings Freund Publishing House Ltd* 1993: 489-492.

11. Haarbrandt B, Schreiweis B, Rey S, et al. HiGHmed - An Open Platform Approach to Enhance Care and Research across Institutional Boundaries. *Methods Inf Med*. 2018;57(S 01):e66-e81. doi:10.3414/ME18-02-0002

12. Eisenhauer EA, Therasse P, Bogaerts J, et al. New response evaluation criteria in solid tumours: revised RECIST guideline (version 1.1). *Eur J Cancer*. 2009;45(2):228-247. doi:10.1016/j.ejca.2008.10.026

13. Hübner G, Neben K, & Stöger H. CUP Syndrom – Krebserkrankungen mit unbekanntem Primärtumor. *DGHO Leilinie 2018*.

14. Bochtler T, Krämer A. Does Cancer of Unknown Primary (CUP) Truly Exist as a Distinct Cancer Entity?. *Front Oncol.* 2019;9:402. Published 2019 May 17. doi:10.3389/fonc.2019.00402

15. Pentheroudakis G, Briasoulis E, Pavlidis N. Cancer of unknown primary site: missing primary or missing biology?. *Oncologist.* 2007;12(4):418-425. doi:10.1634/theoncologist.12-4-418

16. Lin WC, Tsai, CF. Missing value imputation: a review and analysis of the literature (2006–2017). *Artificial Intelligence Review* 2020;53(2): 1487-1509.

17. Austin PC, White IR, Lee DS, van Buuren S. Missing Data in Clinical Research: A Tutorial on Multiple Imputation. *Can J Cardiol.* 2021;37(9):1322-1331. doi:10.1016/j.cjca.2020.11.010

18. Kang H. The prevention and handling of the missing data. *Korean J Anesthesiol.* 2013;64(5):402-406. doi:10.4097/kjae.2013.64.5.402

19. European Medicines Agency. External guidance on the implementation of the European Medicines Agency policy on the publication of clinical data for medicinal products for human use. *EMA/90915/2016 Version 1.4* 2018.

20. Jakob CEM, Kohlmayer F, Meurers T, Vehreschild JJ, Prasser F. Design and evaluation of a data anonymization pipeline to promote Open Science on COVID-19. *Sci Data.* 2020;7(1):435. Published 2020 Dec 10. doi:10.1038/s41597-020-00773-y

21. Malin B, Loukides G, Benitez K, Clayton EW. Identifiability in biobanks: models, measures, and mitigation strategies. *Hum Genet.* 2011;130(3):383-392. doi:10.1007/s00439-011-1042-5

22. Prasser, F., Eicher, J., Spengler, H., Bild, R., & Kuhn, K. A. (2020). Flexible data anonymization using ARX—Current status and challenges ahead. *Software: Practice and Experience*, *50*(7), 1277-1304.

23. Drepper, J (Ed.). IT-Infrastrukturen in der patientenorientierten Forschung: aktueller Stand und Handlungsbedarf 2015. *Akademische Verlagsgesellschaft AKA* 2016.

24. Pedregosa F, Varoquaux G, Gramfort A, et al. Scikit-learn: Machine learning in Python. *the Journal of machine Learning research* 2011; 12: 2825-2830.

25. Hastie T, Tibshirani R, Friedman JH, Friedman, JH. The elements of statistical learning: data mining, inference, and prediction. *Springer*; 2009.

26. James G, Witten D, Hastie T, Tibshirani R. An introduction to statistical learning. *Springer*; 2013.

27. Breiman L. Random forests. *Machine learning* 2001; *45*: 5-32.

28. Friedman JH. Greedy function approximation: a gradient boosting machine. *Annals of statistics* 2001: 1189-1232.

29. Louppe G, Wehenkel L, Sutera A, Geurts P. Understanding variable importances in forests of randomized trees. *Advances in neural information processing systems* 2013; *26*.

30. Krämer A, Bochtler T, Pauli C, et al. Cancer of unknown primary: ESMO Clinical Practice Guideline for diagnosis, treatment and follow-up. *Ann Oncol*. 2023;34(3):228-246. doi:10.1016/j.annonc.2022.11.013

31. Seufferlein T, Mayerle J, Böck S, et al. S3-Leitlinie zum exokrinen Pankreaskarzinom – Langversion 2.0 – Dezember 2021 – AWMF-Registernummer: 032/010OL. *Z Gastroenterol.* 2022;60(11):e812-e909. doi:10.1055/a-1856-7346

32. Lee T, Teng TZJ, Shelat VG. Carbohydrate antigen 19-9 - tumor marker: Past, present, and future. *World J Gastrointest Surg.* 2020 Dec 27;12(12):468-490. doi: 10.4240/wjgs.v12.i12.468.

33. Groß S, Follmann M, Blödt S, Galle PR, Malek NP, Bitzer M. Editorial der S3-Leitlinie Diagnostik und Therapie des Hepatozellulären Karzinoms und biliärer Karzinome. *Z Gastroenterol*. 2023;61(4):372-374. doi:10.1055/a-2026-1182

34. Poruk KE, Gay DZ, Brown K, et al. The clinical utility of CA 19-9 in pancreatic adenocarcinoma: diagnostic and prognostic updates. *Curr Mol Med*. 2013;13(3):340-351. doi:10.2174/1566524011313030003

35. Sinn M, Arnold D, Borner M, et al. Biliäre Karzinome - Karzinome der Gallengänge und Gallenblase. *Onkopedia*, Oct. 2021, www.onkopedia.com/de/onkopedia/guidelines/biliaere-karzinome/@@guideline/html/index.html. Accessed 11 Nov. 2023.

36. Schmiegel W, Buchberger B, Follmann M, et al. S3-Leitlinie – Kolorektales Karzinom. *Z Gastroenterol*. 2017;55(12):1344-1498. doi:10.1055/s-0043-121106

37. Hofheinz RD, Arnold D, Borner M, et al. “Kolonkarzinom.” *Onkopedia*, July 2022, www.onkopedia.com/de/onkopedia/guidelines/kolonkarzinom/@@guideline/html/index.html. Accessed 11 Nov. 2023.

38. Kankanala VL, Mukkamalla SKR. Carcinoembryonic Antigen. In: *StatPearls*. Treasure Island (FL): StatPearls Publishing; January 23, 2023.

39. Wang H, Meng AM, Li SH, Zhou XL. A nanobody targeting carcinoembryonic antigen as a promising molecular probe for non-small cell lung cancer. *Mol Med Rep*. 2017;16(1):625-630. doi:10.3892/mmr.2017.6677

40. Pang WW, Abdul-Rahman PS, Wan-Ibrahim WI, Hashim OH. Can the acute-phase reactant proteins be used as cancer biomarkers?. *Int J Biol Markers*. 2010;25(1):1-11. doi:10.1177/172460081002500101

41. Hart PC, Rajab IM, Alebraheem M, Potempa LA. C-Reactive Protein and Cancer-Diagnostic and Therapeutic Insights. *Front Immunol*. 2020;11:595835. Published 2020 Nov 19. doi:10.3389/fimmu.2020.595835

42. Erdmann, F, Spix, C, Katalinic, A, et al.. Cancer in Germany 2017/2018. Robert Koch-Institut; 2022: 86–89.

43. Miao H, Verkooijen HM, Chia KS, et al. Incidence and outcome of male breast cancer: an international population-based study. *J Clin Oncol*. 2011;29(33):4381-4386. doi:10.1200/JCO.2011.36.8902
